# Immunomodulatory activity of *Buchholzia coriacea* seed methanol extract on *Trypanosoma brucei brucei* infected mice

**DOI:** 10.1080/13880209.2016.1265988

**Published:** 2016-12-12

**Authors:** James I. Eze, Chioma F. Ekelozie, Nwakego E. Nweze

**Affiliations:** Department of Veterinary Medicine, University of Nigeria, Nsukka, Nigeria

**Keywords:** Delayed hypersensitivity reaction, antibody response, trypanosomosis

## Abstract

**Context:** The seeds of *Buchholzia coriacea* Engler (Capparaceae) are used in Eastern Nigeria to treat feverish conditions, and to treat malaria and sleeping sickness that cause fever.

**Objective:** The current study assesses the immunomodulatory activity of *Buchholzia coriacea* seed extract on *Trypanosoma brucei brucei* infected mice.

**Materials and methods:** Delayed hypersensitivity reaction, humoral antibody response and *in*-*vivo* leucocyte mobilization tests were assessed in three different experiments to determine the effect of the extract on immune response. Seventy-five (75) mice (25 mice per experiment) were used for the study and were each infected with 1.00 × 10^6^ trypanosomes intra-peritoneally. Groups A, B and C were given 250, 500 and 1000 mg/kg of the extract, respectively, group D received 7.5 mg/kg body weight of levamisole and group E was the control. Sheep RBCs were used as antigen.

**Results:** The acute toxicity tests did not cause clinical signs or death within 24 h post treatment at all the doses tested. The extract inhibited delayed hypersensitivity reaction by 20.9 and 20.8% at 250 and 500 mg/kg, respectively, while at 1000 mg/kg, the paw size increased (−101.9%) when compared with the control. The extract elevated the antibody titre from 1.60 ± 0.40 for control to 8.00 ± 3.58 for 500 mg/kg group. The extract increased in total leucocytes counts.

**Discussion and conclusion:** The extract has a very wide safety margin and was able to improve immune response. The results of the present study showed that *Buchholzia coriacea* seed methanol extract possesses immunostimulatory activity on trypanosome-infected mice.

## Introduction

The immune system has a fundamental role in protecting the body against pathogenic microbial agents (Amirghofran 2012). It is a part of the body that detects the pathogen by using a specific receptor, and produces immediate response by the activation of immune component cells, cytokines, chemokines and also release of inflammatory mediators (Kumar et al. 2011). The immune system can be manipulated by the use of immunomodulators in disease conditions by achieving immunostimulation (as in the treatment of AIDS) or immunosuppression [suppression of normal or excessive immune function (e.g. the treatment of graft rejection or autoimmune disease)] (Alamgir & Uddin 2010).

Animal trypanosomosis constitutes a major threat to food security in several parts of sub-Saharan Africa including Nigeria (Onyiah 1997; Budd 1999; Swallow 2000). Food and Agriculture Organization (FAO 2008) reported that almost more than any other disease affecting both people and livestock, trypanosomosis straddles the ground between human health, livestock health, agricultural production and rural development; consequently, tackling trypanosomosis has the potential to impact on all 8th (eight) millennium development goals of the FAO which includes eradication of extreme poverty (Ameen et al. 2008). Direct losses due to trypanosomosis are estimated to between US $1 and 1.2 billion each year whereas the indirect impact of African animal trypanosomosis (AAT) on agriculture in sub-Saharan Africa exceeds this amount (Ilemobade 2009; Kumar et al. 2011; Eze & Okonkwo 2013).

Modulation of immune response to alleviate disease conditions has long been of interest and increasingly recognized as a key component of effective disease control. Plant extracts have been widely investigated in the recent time in different parts of the world for their possible immunomodulatory properties (Kumar et al. 2011; Eze & Ndukwe 2012).

Since most of the drugs currently available for treatment of African trypanosomosis are toxic, costly and no longer effective (Eze et al. 2015), attempts are being made in laboratories around the world to discover new, safer, more cost effective and more potent molecules from medicinal plants with an ethnomedical history. The ethanol extract of *Buchholzia coriacea* Engler (Capparaceae) seed was shown to have antitrypanosomal activity in mice experimentally infected with *Trypanosoma brucei* (Nweze et al. 2009) in the experiment. The plant has also been shown to possess antiplasmodial activity (Okoli et al. 2010), antibacterial activity (Mbata et al. 2009), larvicidal effect (Adediwura et al. 2011), antispasmodial and anti-diarrheic properties (Anowi et al. 2012), analgesic activity (Ezeja et al. 2011) and anthelminthic activity (Nweze & Asuzu 2009; Adediwura et al. 2011). The biological activities and mode of action of this plant extracts are poorly understood and may act directly or indirectly.

Phytochemical constituents of *Buchholzia coriacea* include alkaloids, anthraquinones, cardiac glucosides, flavonoids, saponins and tanins (Adediwura et al. 2011; Enechi et al. 2011). In this view, many plant extracts with immunomodulatory and antioxidant activities can be of great help in the control of trypanosomosis. Plants such as *Caesalpinia bonducella* Flem (Caesalpiniaceae)*, Rhododendron spiciferum* Franch (Ericaceae)*, Curcuma longa* Linn (Zingiberaceae) *Azadiracta indica* A. Juss (Meliaceae)*, Boerhaavia diffussa* Linn (Nyctaginaceae) and *Ocimum sanctum* Linn (Lamiaceae) among others, are known to possess immunomodulatory activity (Mahima et al. 2012). Research has shown that some immunomodulators and antioxidants are known to be beneficial in control of trypanosome infection (Amirghofran 2012).

This work was therefore designed to study the immunomodulatory activity of methanol extract of *Buchholzia coriacea* seeds on *Trypanosoma brucei* infected mice with the aim of having a better understanding of the antitrypanosomal activity of the extract.

## Materials and methods

### Plant

*Buchholzia coriacea* is an evergreen tree that grows 30–60 ft high in the forest. It has a deep red slash, cream-coloured flowers, fruits are yellowish when ripe, blackish seeds and is spicy when tasted. Common names are – Wonderful cola, Musk tree and Elephant kola. It is found in Eastern and Western Nigeria, extending from the Ivory Coast to Gabon (Keay et al. 1964). It was named after Buccholz who collected the plant in the Cameroons in the late nineteenth century.

### Experimental animals

A total of 75 adult male out-bred albino mice weighing 23–28 g were used for the study. The animals were housed in a fly proof laboratory animal house and given pelleted chick grower feed and water *ad libitum*. Animal studies were in compliance with the ethical procedures of the Animal Use and Care Committee, Faculty of Veterinary Medicine, University of Nigeria, Nsukka (number: EC/2015 – 0028) which corresponds with National Institutes of Health (NIH) guidelines (NIH 1996).

### Trypanosome parasite

*Trypanosoma brucei brucei* was used for this study. It was isolated from a clinically sick dog. The trypanosome was maintained in the Department by serial passages in mice. Trypanosoma identification was by morphology characteristics of the parasites in the stained smears. The examination of all the samples was made with oil immersion microscope (Murray et al. 1983). The parasite was identified to sub-specie level using Blood Incubation Infectivity Test (BIIT) of Rickman and Robson (1970).

### Preparation of the plant extract

The mature seeds of *B. coriacea* Engler were collected from Nsukka town in February, 2009 and identified by a taxonomist Mr A. O. Ozioko of the Bioresources Development and Conservation Centre (BDCP), Nsukka where voucher specimens were also deposited. Ground *Buchholzia coriacea* seeds were extracted according to Alanis et al. (2005). The seeds were pulverized into fine powder in a mill. The powdered plant materials were stored in sealed cellophane bags in order to protect them from light. In the methanol extract (70%), nitrogen gas was used to evaporate the solvent. To further ensure that all the water was removed, the extract was freeze dried (Edward’s high vacuum Crawley, England). The extract was stored at 4 °C until use.

### Acute toxicity test

In the acute toxicity test, graded doses (250, 500, 1000, 2000 mg/kg) of the extract was administered intra-peritoneally to four groups of six mice each. They were monitored for acute toxicity signs such as behavioural changes or death for a period of 24 h (Nweze et al. 2009).

### Technique for estimating trypanosomes

This technique was described by Herbert and Lumsden (1976). A drop of blood from the tail of a mouse was examined under 40 × magnification of a table microscope and the number of trypanosomes in a field was matched with the reference table. The log figures were converted to absolute numbers which reflected the number of trypanosomes per millilitre.

### Red blood cell antigen

Fresh sheep red blood cells (SRBCs) used as antigens were obtained from blood collected by venipuncture of a West African dwarf sheep. Sheep blood (3 mL) was collected. Before use, the red blood cells were washed three times with 15 mL of phosphate buffered saline (PBS), pH 7.2, by centrifugation at 3000 × *g* for 10 min on each occasion using desktop centrifuge. After the final wash, the SRBSCs were suspended in PBS as a 2% suspension (based on packed cell volume) for the serological tests and as a 10% suspension for immunization of the mice. A 1 mL amount of the 2% suspension contained approximately 10^9^ red blood cells (Nworu et al. 2007).

### Delayed hypersensitivity reaction

Delayed hypersensitivity reaction was induced in mice using SRBCs as antigen (Nworu et al. 2007). Twenty-five adult male mice divided into five groups of five mice each were used for the study. The mice were infected with 1.00 × 10^6^*Trypanosoma b. brucei* intra-peritoneally. On day 7 post-infection, groups A, B and C were given 250, 500 and 1000 mg/kg of the extract, respectively, while group D received 7.5 mg/kg body weight of Levamisole and group E left untreated as control sheep RBCs (0.2 mL of 10^9^ cells mL) were administered (subcutaneous) on the planter region of right hind foot pad on day 0 to sensitize the animals. On day 5, subcutaneous injection of the same amount of antigen was administered in the left hind pad as a challenge. Edema was produced by antigenic challenge in the left foot pad and was measured as the difference in the paw thickness before and 24 h after the challenge. This was done with a vernier caliper (Naved et al. 2005). *Buchholzia coriacea* (250, 500 and 1000 mg/kg) was administered 3 days prior to sensitization, and continued until challenge (Naved et al. 2005). Also, the parasitaemia were estimated the day the administration of *B coriaca* started, and again on the day the administration stopped 8 days later, using Herbert and Lumsden (1976) method.

### Humoral antibody response

Twenty-five adult male mice divided into five groups of 5 mice each were used for the study. The mice were infected with 1.00 × 10^6^*Trypanosoma b. brucei* intra-peritoneally. On day 7 post-infection, groups A, B, C were given 250, 500 and 1000 mg/kg of the extract, respectively, while group D received 7.5 mg/kg body weight of levamisole and group E was left as a control. Sheep RBCs (0.1 mL^−^^1^ of 10^9^ mL^−^^1^) were used to immunize by intra-peritoneal injection on day 0 and challenged by similar IP injection of the same amount in day 5 post-immunization (PI). Primary antibody titre on day 10 PI by the hemagglutination technique (Nelson & Mildenhall 1967).

### *In vivo* leucocytes mobilization

Leucocytes mobilization as described by the method of Ribeiro et al. (1991) was used to study the effect of the *Buchholzia coriacea* extract on *in vivo* leucocytes migration induced by inflammatory stimulus. Twenty-five adult male mice divided into five groups of 5 each were used for the study. The mice were infected with 1.00 × 10^6^*Trypanosoma b. brucei* intraperitoneally. On day 7 post infection, groups A, B and C were given oral administration of 250, 500 1000 mg/kg weight of extract, respectively, while group D received 7.5 mg/kg body weight of Levamisole and group E was left as a control. One hour later, each mouse in groups A, B, C, D and E received intraperitoneal injection of 0.5 mL of 3% agar suspension in normal saline. Four hours later, the mice were sacrificed under anaesthesia and the peritoneum washed with 5 mL of phosphate buffer saline containing 0.5 mL of 10% EDTA. The peritoneal fluid was recovered and total leucocytes counts (TLC) determined with haemocytometer and the differential cell count was determined by microscopic counting of Giemsa stained perfusate smear on glass slide.

### Statistical analysis

Results were analyzed using one way Analysis of Variance (ANOVA; Fischer LSD *post hoc* test) and expressed as mean ± standard error of mean. Differences between means of treated and control groups were considered significant at *p* < 0.05.

## Results

The result of the acute toxicity tests showed that the extract did not cause clinical signs or death within 24 h post-treatment at all the dose levels tested. The LD_50_ is therefore greater than 1000 mg/kg. The extract was able to inhibit delayed hypersensitivity reaction at 250 and 500 mg/kg (Table 1). However, at 1000 mg/kg, the paw size increased significantly when compared with the control. The levamisole-treated group showed no difference from the infected control. The extract was able to reduce the parasite level in the treated groups with the 500 and 1000 mg treated group being significantly (*p* ≤ 0.01) lower than other groups (Table 2). The extract caused elevation of secondary sheep RBCs specific antibody titre (Table 3) at all doses of the extract tested. The increased antibody titre was not dose dependent. The elevation was more pronounced at 500 mg/kg than in 250 and 1000 mg/kg. However, all the extracts treated groups were significantly higher than the control but not with the Levamisole-treated group. The effect of the extract on *in vivo* leucocytes mobilization led to increase in total leucocytes count. The 1000 mg/kg produced the highest leucocytes mobilization and was significantly higher than levamisole and control group. In differential leucocytes mobilization, all the tested doses improved the neutrophil and lymphocyte counts. However, the basophil, monocyte and eosinophil of the extract treated group showed decrease in number when compared with the control group (Table 4).

**Table 1.** Effect of methanolic extract of *Buchholzia coriacea* seeds on delayed hypersensitivity reaction mice estimated as paw swelling (mm).

Treatment	Dose (mg/kg)	Paw swelling (mm)	Inhibition (%)
Extract	250	0.84 ± 0.22^a^	20.9
Extract	500	0.84 ± 0.07^a^	20.8
Extract	1000	2.14 ± 0.02^b^	−101.9
Levamisole	7.5	1.06 ± 0.09^a^	0
Control	–	1.06 ± 0.09^a^	–

Mean ± SEM with different superscript letters are significantly different (*p* < 0.05).

**Table 2.** Effect of methanolic extract of *Buchholzia coriacea* seeds on the parasitaemia (10^6^ parasites/ml) before and after treatment.

Treatment	Dose (mg/kg)	Day 0 of the treatment Parasites (10^6^)	Day 8 of the treatment Parasites (10^6^)
Extract	250	22.38 ± 42321.09	28.32 ± 43425^a^
Extract	500	19.02 ± 81782.12	18.09 ± 109934^b^
Extract	1000	20.87 ± 11223	16.82 ± 98852^b^
Levamisole	7.5	34.98 ± 87822.65	35.12 ± 21345^a^
Control	–	29.23 ± 34245	89.93 ± 23424^c^

Mean ± SEM with different superscript letters are significantly different (*p* < 0.05).

**Table 3.** Effect of methanolic extract of *Buchholzia coriacea* seeds on primary and secondary antibody response (log_10_2) in mice.

Treatment	Dose (mg/kg)	Primary antibody	Secondary antibody
Extract	250	3.20 ± 1.36	3.20 ± 1.36^a^
Extract	500	3.20 ± 0.49	8.00 ± 3.58^a^
Extract	1000	5.60 ± 2.64	6.80 ± 2.50^a^
Levamisole	7.5	2.80 ± 0.80	6.80 ± 3.77^a^
Control	–	1.20 ± 0.49	1.60 ± 0.40^b^

Mean ± SEM with different superscript letters are significantly different (*p* < 0.05).

**Table 4.** Effect of *Buchholzia coriacea* seed extract on total and differential leucocytes mobilization (cells/ml) in mice.

	250 (mg/kg)	500 (mg/kg)	1000 (mg/kg)	Levamisole	Control
TLC	1450.00 ± 0.22	1430 ± 367.29	1670 ± 371.01	1110.00 ± 167.63	950.00 ± 184.3
NEUT	279.80 ± 70.59	362.60 ± 104.53	397.40 ± 84.72	277.80 ± 69.35	182.00 ± 733.40
LYMPH	1149.40 ± 369.63	1042.80 ± 265.98	1303.80 ± 100.52	826.40 ± 103.85	733.40 ± 144.79
BASO	5.80 ± 5.80^a^	0.00 ± 0.00^a^	0.00 ± 0.00^a^	1.60 ± 0.60^a^	16.60 ± 3.49^b^
MONO	10.20 ± 2.34^ab^	1.40 ± 0.40^a^	17.80 ± 4.91^b^	1.60 ± 0.60^a^	18.00 ± 3.27^b^
EOSIN	0.00 ± 0.00	5.80 ± 0.50	5.80 ± 0.55	0.00 ± 0.00	6.40 ± 2.91

Mean ± SEM with different superscript letters are significantly different (*p* < 0.05).

## Discussion

The result of the acute toxicity tests showed that the extract has a very wide safety margin. This is an indication that the extract was well tolerated by mice. This was in agreement with earlier work done by Nweze et al. (2009). The delayed hypersensitivity reaction was inhibited by the extracts at dose of 250 and 500 mg/kg. The ability of the extract to inhibit the delayed hypersensitivity reaction in this experiment may be due to its anti-inflammatory properties. This reaction is mediated by T cells and monocytes/macrophages rather than by antibodies. Delayed hypersensitivity reaction is a major mechanism of defence against various intracellular pathogens, including Mycobacteria, fungi and certain parasites and it occurs in transplant rejection and tumour immunity (Vojdani et al. 2008; Singhal & Ratra 2013). The term “Delayed” is used to differentiate a secondary cellular response which appears 48–72 h after antigen exposure from an immediate hypersensitivity response which generally appears within 12 min of an antigen challenge. DTH requires specific recognition of the given antigen by activated T lymphocytes which subsequently proliferate and release cytokines (Singhal & Ratra 2013). These will in turn increase vascular permeability and activation, promoting increased phagocytic activity and increased concentration of lytic enzymes for effective killing (Descotes 1999; Kannan et al. 2009). This could be responsible for the increase in paw size (erythema) as seen in 1000 mg/kg. The increase noticed at 1000 mg was an indication that the extract may have both pro- and anti-inflammatory properties, which depends on the dose administered. The ability of the extract to reduce the parasite level was in agreement with work done by Nweze et al. (2009) on *T. brucei* but at variance with report by Nweze et al. (2011) using *T. congolense*. The ability of the extract to reduce the parasitaemia in this experiment could be attributed to prolonged administration and its immunomodulatory effect.

The sheep RBCs antibody titre was elevated in all extract-treated groups. Effect of enhancement of the antibody production in this experiment may be associated with effect of the extract on lymphoid organs. When the mice were sensitized with the sheep RBCs, SRBC antigen was taken up by macrophages and was processed. When a T lymphocyte sees the processed antigens on the B cell, the T cell stimulates the B cells to undergo repeated cell divisions, enlargement and differentiation to form a clone of antibody secreted by plasma cells (Stewart & Weir 1997). Hence, the antibody then binds to the antigen, making them easier to ingest by the white blood cells. Antibodies are glycoproteins belonging to the immunoglobulin super family; they are typically made of basic structural units each with two large heavy chains and two small light chains (Butler 1998). Antibodies can occur in two physical forms (Sheehan 2002): a soluble form that is secreted from the cell, and a membrane bound form that is attached to the surface of B cells. This is referred to as the B cell receptor (BCR) and is only found on the surface of B cells. It facilitates the activation of these cells and their subsequent differentiation into either antibody factories called the plasma cells or memory B cells which survive in the body and remain that same antigen, so the B cells can respond faster upon exposure (Butler 1998). Soluble antibodies are released into the blood and tissue fluids as well as many secretions to continue to survey for invading microorganisms. This indicates enhanced responsiveness of macrophages, T and B lymphocytes involved in antibody synthesis (Sheehan 2002). The present study showed that the extract increased the total leucocytes count of the perfusate when compared with the control group. Leucocytes migration is important for the transport of immunological information between different compartments of the immune system (Förster & Sozzani 2013). In the current study of the differential leucocytes mobilization, all the tested doses improved the neutrophil and lymphocyte counts. This result may be due to an enhanced immune system. Neutrophils are normally found in the blood stream and in the beginning phase of acute inflammation particularly as a result of bacterial infection, environmental exposure and some cancers. Neutrophils are one of the first responders of inflammatory cells to migrate towards the site of inflammation (Kumar & Sharma 2010). They migrate through the blood vessels, and then through interstitial tissue, following chemical signals such as interleukin-8, C5a, etc. in a process called chemotaxis. Neutrophils are recruited to the site of injury within minutes following trauma and are the hallmark of acute inflammation (Jones et al. 2005). The basophils, monocytes and eosinophils of the extract-treated group showed decrease in number when compared with the control group. These cells are more sensitive to the effects of immunomodulatory cytokines and pro-inflammatory factors, which are likely to be in abundance in inflammatory conditions (Pecaric-Petkovic et al. 2009). Basophils, eosinophils, and Th2 lymphocytes are also recruited to the site of inflammation and are direct leukocyte targets of IL-33, suggesting a prominent role for these cells of the innate immune system in the biology of IL-33 (Falcone et al. 2000). Basophils play a potentially supportive role in anti-parasite immune responses through their activation by immune serum and production of cytokines (Sullivan et al. 2011).

The result of the current study suggests that immunomodulation may be a key factor in antitrypanosomal activity of methanol extract of *Buchholzia coriacea* seeds.
